# Evaluation of Truview evo2^®^ Laryngoscope In Anticipated Difficult Intubation – A Comparison To Macintosh Laryngoscope

**Published:** 2009-04

**Authors:** Ishwar Singh, Abhijit Khaund, Abhishek Gupta

**Affiliations:** 1 Chairman and Head, Department of Anaesthesiology and Critical Care, Jaipur Golden Hospital, New Delhi; 2 Junior Consultant, Department of Anaesthesiology and Critical Care, Jaipur Golden Hospital, New Delhi; 3 P.G.Student, Department of Anaesthesiology and Critical Care, Jaipur Golden Hospital, New Delhi

**Keywords:** Difficult airway, Truview laryngoscope, Macintosh laryngoscope

## Abstract

**Summary:**

The aim of the study was to assess and compare laryngoscopic view of Truview evo2 laryngoscope with that of Macintosh laryngoscope in patients with one or more predictors of difficult intubation (PDI). Moreover ease of intubation with Truview evo2 in terms of absolute time requirement was also aimed at. Patients for elective surgery requiring endotracheal intubation were initially assessed for three PDI parameters – modified Mallampati test, thyro-mental distance & Atlanto-occipital (AO) joint extension. Patients with cumulative PDI scores of 2 to 5 (in a scale of 0 to 8) were evaluated for Cormack & Lehane (CL) grading by Macintosh blade after standard induction. Cases with CL grade of two or more were further evaluated by Truview evo2 laryngoscope and corresponding CL grades were assigned. Intubation attempted under Truview evo2 vision and time required for each successful tracheal intubation (i.e. tracheal intubation completed within one minute) was noted. Total fifty cases were studied. The CL grades assigned by Macintosh blade correlated well with the cumulative PDI scores assigned preoperatively, confirming there predictability. Truview evo2 improved laryngeal view in 92 % cases by one or more CL grade. Intubation with Truview evo2 was possible in 88% cases within stipulated time of one minute and mean time of 28.6 seconds with SD of 11.23 was reasonably quick. No significant complication like oro- pharyngeal trauma or extreme pressor response to laryngoscopy was noticed. To conclude, Truview evo2 proved to be a better tool than conventional laryngoscope in anticipated difficult situations.

## Introduction

**Fig 1:**
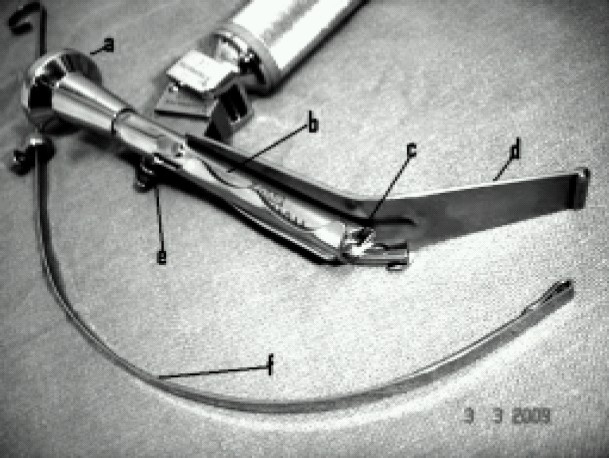
Truview evo2 with preformed stylet- a. View piece; b. Optical tube; c. Prism; d. Blade with distal curved portion; e. Oxyport; f. preformed stylet.

Truview laryngoscope blade (Fig 1) has been developed by Truphatek International^®^ of Israel as an alternative to currently used conventional laryngoscopes to overcome their shortcomings in difficult intubation (DI) situations. Truview has an attached optical assembly based on prism principle to provide image of an object situated at an angle to straight line of vision. Hence Truview should be able to view glottic structures normally not visible to naked eye vision under direct laryngoscopy.

Moreover it has other added features like compatibility to endoscopic camera for enlarged view on a monitor, oxyport to provide continuous oxygen insufflation and fibreoptic light channel. The evo2 version was launched in 2004. But it still awaits sufficient clinical evaluation and critical acclaim by end users.

Li et al (2007)^1^ in their study compared Cormack & Lehane (C&L) grade by Truview and Macintosh blade and their respective intubation times in a series of two hundred cases. They confirmed improved laryngeal view with Truview evo2 although it took slightly longer time to intubate with the device as compared to Macintosh blade. The investigators started the study with individual experience with Truview evo2 of twenty cases or more. The study did not consider patients with predictors of difficult intubation exclusively. But individual case reports of its usefulness in difficult situations are available^2^.

The main objective of our study was to get experience of Truview evo2 in cases with single or multiple predictors of difficult intubations. While choosing predictors of difficult intubations (PDI) we mainly focused on two major anatomical factors that limit conventional laryngoscopy^3^ –

- Disproportionately large tongue base as compared to pharyngeal size – routinely assessed by Mallampati test (modified).
- Difficulty in achieving oro- pharyngo- laryngeal alignment – either due to limitation of atlanto-occipital joint (AO joint) movement or due to reduced man-dibular space (usually assessed by measurement of thyro-mental or thyrohyoid distance etc).

We have chosen three parameters of PDI–Mallampatti Test, AO joint extension & thyro-mental distance to maintain simplicity of assessment and at the same time to ensure predictability. Most multivariate models included these parameters along with few variations as they have high sensitivity and reasonably good specificity^4^–^6^.

We have undertaken the study after gaining cumulative experience in more than two hundred cases in both routine and difficult situations over a period of one year so that our subjective findings get more appealing to the clinical community.

## Methods

**Fig 2:**
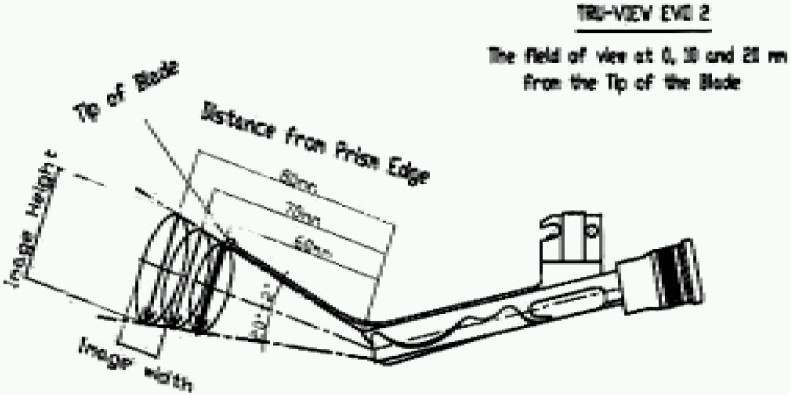
Showing Truview evo2 blade with optical assembly and its image height and width at an angular location at different distances from the tip of the blade

**Fig 3:**
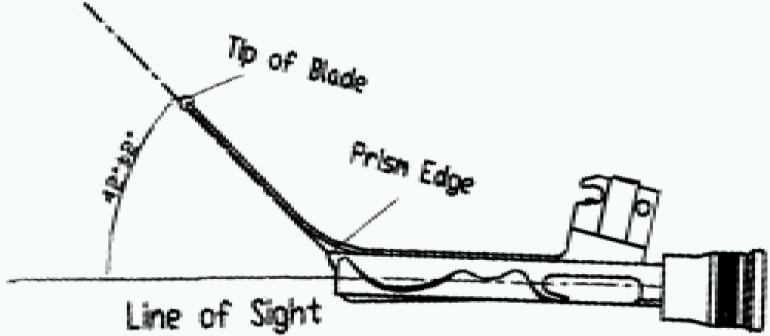
Deviation of angle of vision from straight line of sight due to prism effect of Truview evo2

Truview evo2^®^ laryngoscope was developed by Truphatek International^®^ in 2004. The blade has been designed to accommodate a tubular optical assembly in a groove on its dorsal surface (Fig 1). The optical tube uses prism to produce anterior refraction of more than 35° and thereby enables the viewer to get images of objects situated more anterior to actual line of vision (Fig 2 & 3). The proximal view piece of the assembly is also compatible with endoscopic camera and normally unmagnified image of the scope can be viewed in the monitor with magnification and clarity.

In our study we used Truview blade of adult size and Macintosh blade no 3 & 4 as deemed appropriate by the laryngoscopists. Karl-Storz endoscopic camera was used with Truview evo2 to get magnified monitor view.

After obtaining institutional ethics committee approval and written informed consent from each individual, patients of either sex, within the age group of 20 to 60 years, ASA grade 1 or 2 undergoing elective surgery requiring general anaesthesia and endotracheal intubation were considered for study. The study was conducted at Jaipur Golden Hospital, New Delhi, India in the period between February 2006 and May 2007. Exclusion criteria included patients with a oropharyngeal or laryngeal pathology, cervical trauma, raised intra cranial pressure, head & neck pathology, high risk for gastric content aspiration and patients with anticipated difficulty in bag and mask ventilation (morbid obesity).

All patients were evaluated for 3 predictors of difficult intubation -

- Modified Mallampati test in sitting position with fully protruded tongue.
- Thyro-mental distance (TM distance) in cm distance from the mentum to the thyroid notch while patient's neck is in full extension.
- Atlanto –occipital joint extension in degree – recorded by measuring the angular distance traveled by occlusion surface of the upper incisors while achieving full extension from neutral position. Goniometry was done by plastic caliper type goniometer.

Numerical scores were assigned for each grade or class of difficulty in each category as per Table 1. Cumulative PDI score of 8 predicted maximum difficulty and score of 0 indicated none. We included cases with minimum PDI score of 2 and excluded those with score more than 5.

**Table 1:** Numerical scores for individual predictors of Difficult intubation

Parameter	Difficulty grade/class	Numerical scores
Mallampatti(Modified)	Grade I	0
	Grade II	1
	Grade III	2
	Grade IV	3
Atlanto occipital joint extension	Grade I (> 35°)	0
	Grade II(22° -34°)	1
	GradeIII (12°-21°)	2
	Grade IV(<12°)	3
Tyromental distance	Grade I(>6.5cm)	0
	Grade II (6-6.5cm)	1
	Grade III(<6cm)	2

Selected cases underwent routine pre anaesthetic check up and laboratory investigations as per institutional protocol. Eight hours of fasting was recommended and pre medication with oral diazepam 5 mg one hour prior to surgery was prescribed to all cases. In the operating room standard monitoring was employed. Induction was done with propofol 1.5 to 2mg .kg^−1^ as sufficient to abolish eyelash reflex, fentanyl 1.5 mcg.kg^−1^, and midazolam 0.02 mg.kg^−1^ after 3 minutes of preoxygenation with 100% oxygen. Patients were then assessed for ease of bag and mask ventilation and those whom could not be ventilated with ease were excluded from the study. Rest of the patients were paralyzed with succinylcholine 1.5 mg.kg^−1^. Laryngoscopy was done after 90 seconds. Patient's head was placed on an 8 cm high cushion and manipulations to achieve maximum possible sniffing position were done. Cormack & Lehane grade with Macintosh laryngoscope was recorded in this position by one of the two laryngoscopists involved in the study using either a no 3 or a no 4 blade as deemed suitable by him. No external laryngeal manipulation or aid was taken to improve C&L grade. Cases with C&L grade two or more were further evaluated by Truview. A second laryngoscopist performed Truview evo2 laryngoscopy with endoscopic camera attached to the view piece. He was kept blind of the finding of the first laryngoscopist. The C&L grade by Truview evo2 was noted from monitor view. Endotracheal tube either no. 7 or no. 8 (Portex, cuffed), depending on female or male patient, mounted on a stylet with preformed curve (as provided by Truview manufacturers) was negotiated under Truview vision. Any attempt requiring more than one minute time was terminated as failed attempt and alternative method for tracheal intubation applied immediately. Time taken for intubation was noted (as the time from passing the tip of the laryngoscope blade through the incisor gap till appearance of capnographic tracing). Laryngoscopy was done by two laryngoscopists having more than fifteen years of experience with conventional laryngoscoy and more than one year of experience with Truview evo2. In every alternate case the laryngoscopists switched role with the instruments. Each did 25 cases with Macintosh and 25 cases with Truview Laryngoscope. Total 53 cases were assessed with Macintosh blade and 3 cases were subsequently excluded due to recorded C & L grading of one. Rest 50 cases (with CL grade two or more by Macintosh) completed the whole evaluation process.

Data were analyzed using Wilcoxon test and Spearman rank test, and linear regression to determine association between airway parameters and laryngoscopic findings.

## Results

Demographic profiles of the patients are shown in Table 2. Both male and female patients are adequately represented. Average age of the patients is 41.8 years (SD 13).

**Table 2:** Demographic profile of the study group

Patient characteristics	Ratio or Mean (SD)
Sex, M: F	23:27
Age(Yrs)	41.8(13)
Height (cm)	161.3(9.6)
Body Weight (Kg)	60.5(9.75).

Table 3 shows the C & L gradings by Macintosh laryngoscope as per the different PDI scores assigned preoperatively. PDI scores were found to be significantly related to C & L gradings by Macintosh blade. Three cases with PDI score of 2 had C&L grade I and subsequently ruled out from further evaluation. Rest fifty cases with C&L grade II or above were further evaluated with Truview evo2 and corresponding C&L grades are plotted in the Table 4.

**Table 3:** C & L grade by Macintosh laryngoscope as per the PDI scores

C&L by Macintosh	PDI scores				Total (n = 53)
	2 (n = 20)	3 (n = 19)	4 (n = 11)	5 (n = 3)	
Grade I	3				3
GradeII	7	6	3	0	16
Grade III	10	13	5	0	28
Grade IV	0	0	3	3	6

**Table 4:** C & L grade by Truview evo2 for each grade by Macintosh laryngoscope

C&L by Truview evo 2	C&L by Macintosh			Total (n =50)
	GradeII (n = 16)	GradeIII (n = 29)	GradeIV (n = 5)	
Grade I	15	21	3	39
GradeII	1	5	2	8
Grade III	0	3	0	3
Grade IV	0	0	0	0

46 cases (92%) showed improvement in glottic view by I or II C&L grade while assessed with Truview evo2 (Table-4 & Table-5). 15 out of 16 cases of Macintosh Grade II showed Grade I view by Truview evo2. 21 cases out 29, with Macintosh Grade III had improvement by two grades to Grade I by Truview evo2. All the five cases with Grade IV view by Macintosh were improved to Grade I or II level by Truview evo2. In four cases (i.e. 8%) we did not observed any improvement in view. The differences in view by both the laryngoscopes have been found very strongly significant and consistent for all grades of laryngeal view.

**Table 5:** Time of Tracheal Intubation (TTI) as per C&L grading by Truview evo2

CL grade by Truview	No of successful intubations (percentage)	Mean TTI (± SD) in seconds
I(n = 39)	37 (94)	27.3±12.10
II(n = 8)	6 (75)	31.3±9.00
III(n = 3)	1 (33)	45.0
Total(n = 50)	44(88)	28.6±11.23

Forty four cases out of fifty (88%) could be intubated within the stipulated time of sixty seconds (Table 5). Average time of tracheal intubation (TTI) by Truview evo2 was 28.6 seconds (S.D. 11.23). Mean TTI for cases of C&L grade I&II were 27.3 seconds and 31.3 seconds respectively and did not show statistically significant difference. Of the three cases with grade III view only one could be intubated within sixty seconds

## Discussion

The Truview evo2 laryngoscope has been designed using optical principle to provide better view of objects situated more anterior to straight line of vision. It is deemed to be useful in situations where conventional laryngoscopy fails to get desired laryngeal view. Few trial reports available so far have shown improvement in laryngeal view although intubation under Truview vision took longer time as compared to conventional scopes^1^,^7^. Keeping in view the very limited assessment reports available we have tried to evaluate the instrument in anticipated difficulty assessed by three parameters.

Our results have shown definite improvement in laryngeal view as compared to Macintosh blade. Even the few cases showing grade IV view by Macintosh have been improved to grade I or II view by Truview. The average time taken for negotiation of ETT was much less than reported by other authors^1^,^7^. Considering difficulty involved in all the cases our recorded average time for tracheal intubation seems significantly quicker.

On the other hand tracheal intubation time did not bear much difference whether it was grade I or grade II C&L view. We experienced requirement of certain manipulative movements while negotiating the endotracheal tube under Truview evo2 vision even when the best of the laryngeal view was available. This was the main reason for requirement of longer time for tracheal intubation under Truview vision as compared to conventional laryngoscopy and experienced by other authors also. But it has been able to overcome one of the main disadvantage of earlier prism fitted equipments^8^ i.e. bulkiness obstructing intubation. Hence it is note-worthy that it involves probably a little longer learning curve but becomes handy with repeated use.

Another aspect of Truview evo2 use that we would like to share with readers that fogging of the lens situated at the distal end of the optical tube may blur the vision very often unless it is well taken care of. We successfully overcame this difficulty by warming the distal end of the optical tube by dipping in warm water just prior to laryngoscopy or by the use of continuous flow of oxygen by the oxyport @ 6 L. Min^−1^. Out of the six failed intubations (could not be completed in sixty seconds) three cases had the problem of blurring of view requiring repeated attempt. Rest three cases had short thyro-mental distance and high Mallampati class (PDI 5).

Throughout the trial we did not observe any airway trauma with Truview evo2. Haemodynamic responses to laryngoscopy were within expected limits. To conclude, Truview evo2 proved to be a better device than Macintosh laryngoscope in anticipated difficult situations.
